# Adult Type 3 Adenylyl Cyclase–Deficient Mice Are Obese

**DOI:** 10.1371/journal.pone.0006979

**Published:** 2009-09-11

**Authors:** Zhenshan Wang, Vicky Li, Guy C. K. Chan, Trongha Phan, Aaron S. Nudelman, Zhengui Xia, Daniel R. Storm

**Affiliations:** Department of Pharmacology, University of Washington, Seattle, Washington, United States of America; Medical College of Georgia, United States of America

## Abstract

**Background:**

A recent study of obesity in Swedish men found that polymorphisms in the type 3 adenylyl cyclase (AC3) are associated with obesity, suggesting the interesting possibility that AC3 may play a role in weight control. Therefore, we examined the weight of AC3 mice over an extended period of time.

**Methodology/Principal Findings:**

We discovered that AC3^−/−^ mice become obese as they age. Adult male AC3^−/−^ mice are about 40% heavier than wild type male mice while female AC3^−/−^ are 70% heavier. The additional weight of AC3^−/−^ mice is due to increased fat mass and larger adipocytes. Before the onset of obesity, young AC3^−/−^ mice exhibit reduced physical activity, increased food consumption, and leptin insensitivity. Surprisingly, the obesity of AC3^−/−^ mice is not due to a loss of AC3 from white adipose and a decrease in lipolysis.

**Conclusions/Significance:**

We conclude that mice lacking AC3 exhibit obesity that is apparently caused by low locomotor activity, hyperphagia, and leptin insensitivity. The presence of AC3 in primary cilia of neurons of the hypothalamus suggests that cAMP signals generated by AC3 in the hypothalamus may play a critical role in regulation of body weight.

## Introduction

Based on the definition of obesity, about 65% of the U.S. population is overweight (Body Mass Index >25), and over 30% is obese (Body Mass Index >30) [1]. Obese individual have a higher risk for a number of diseases including type 2 diabetes, cardiovascular disease, metabolic syndrome, hypertension, certain forms of cancer, and sleep-breathing disorders [1]. Furthermore, obesity decreases longevity [2], [3], [4] and lowers the general quality of life [5]. Although intensive effort has been devoted to anti-obesity therapy, the percentage of obese individuals in industrialized countries continues to increase.

Obesity is a disorder of energy balance and it occurs when energy intake persistently exceeds energy expenditure. Although it has been generally accepted that reduced physical activity with a sedentary lifestyle contributes to the increasing prevalence of obesity, the underlying molecular mechanisms are largely unknown. In humans, twin studies show that 78% of the variance in physical activity can be explained by genetic factors [6] and a study with over 4,000 twin individuals showed that physical inactivity in adolescence strongly predicts the risk for obesity in adults [7]. Moreover, studies with monozygotic twin pairs indicate that physical activity, or lack thereof, is both causative and secondary to the development of obesity [7].

Recently it was reported that AC3 gene polymorphisms are associated with obesity in a group of Swedish men [8]. This suggests the interesting possibility that AC3, which is expressed in adipose tissue and the hypothalamus, may play an important role in the regulation of body weight. To test this hypothesis, we monitored the weight of AC3^−/−^ mice over an extended period of time. Here, we report that mice lacking AC3 exhibit obesity which is apparently caused by low locomotor activity, hyperphagia, and leptin resistance.

## Materials and Methods

### Animals

AC3^−/−^ mice and wild type littermates were bred from heterozygotes and genotyped as previously reported [9]. Wild type littermates were used as controls. Mice were maintained on a 12 hr light/dark cycle and had access to water and food *ad libitum*. Except as indicated, all animals were 6 to 12 months old. Animals were housed individually and allowed to habituate in the new cages for at least one week before recording food consumption. Food consumption was measured daily for at least two weeks. During fasting, the animals were without food for 18 hrs. All animal procedures were approved by the Institutional Animal Care and Use Committee at the University of Washington.

### Adenylyl cyclase activity assay

Adenylyl cyclase activity was measured as previously described [10]. Briefly, epididymal white or brown adipose tissue was homogenized in cold buffer containing 25 mM Tris/HCl (ph 7.4), 250 mM sucrose, 1 mM MgCl_2_, and protease inhibitor cocktail tablets (1 tablet/10 ml; Roche, Indianapolis, IN). The homogenate was centrifuged at 1,100×g for 15 min at 4°C, and the supernatant was centrifuged at 48,000×g for one hr to obtain a membrane preparation. Protein concentrations were determined by BCA assay kit (Pierce, Rockford, IL). Adenylyl cyclase activity was measured in a buffer consisting of 1 mM cyclic AMP, 10 mM phosphocreatine, 0.5 unit of creatine phosphokinase, 5 µM GTP, 5 mM MgCl_2_, 0.2 mM EDTA, 50 mM Tris/HCl (pH 7.5), with [α-^32^P] ATP to 5×10^6^ cpm/reaction at 30°C for 15 min.

### Western blot analysis

Western blot analysis was performed as previously described [9]. Isolated brown adipose tissue and white adipocytes were homogenized on ice in homogenization buffer. Protein concentrations were measured with a BCA assay kit (Pierce, Rockford, IL) according to the manufacturer's instructions. Samples (40 µg to 60 µg) were boiled at 95°C for more than 5 min, then placed on ice and loaded onto 7.5% polyacrylamide gels. The gels were transferred to a PVDF membrane (Mllipore), and blocked with 5% milk in phosphate-buffered saline (PBS) with 0.05% Tween 20 for 1∼2 hr. Blots were incubated with polyclonal AC3 antibody (1∶1000; Santa Cruz Biotechnology, Inc.) over night at 4°C, followed by horseradish peroxidase-conjugated goat anti-rabbit lgG for 1 hr at room temperature. Blots were developed with an ECL detection reagent kit (Amersham Biosciences) according to the manufacturer's instructions.

### Histology

Segments of epididymal white adipose tissue from 6- to 8- month old of AC3^−/−^ and AC3^+/+^ mice (each genotype consisted of 2 male and 2 female) were fixed in Bouin's solution (Sigma-Aldrich, St. Louis, MO), dehydrated in ethanol, embedded in paraffin, and cut at a thickness of 5 µm. Sections were deparaffinized, rehydrated, and stained with hematoxylin/eosin.

### Immunofluorescene

Immunofluorescene procedures were performed as described previously [9] with a few modifications. Mice were perfused with a mixture consisting of half 4% paraformaldehyde (PFA) and half HistoChoice (Amresco). The brains were then dissected and post fixed with the same fixing solution overnight at 4°C. Then the brains were cryoprotected with 30% sucrose for 48 hr at 4°C. Brains were cut into 30 µm sections with a cryostat freezing microtome. Floating sections were blocked and then first incubated with the Somatostatin receptor subtype 3 (Sstr3) antibody (1∶5000, Santa Cruz Biotechnology, Inc.) overnight at 4°C and processed with a TSA™ Cyanine 3 system (Perkin Elmer LAS, Inc.) according to the manufacturer's instructions. The sections were incubated with AC3 antibody (1∶500, Santa Cruz Biotechnology, Inc.) overnight at 4°C followed by the secondary antibody, Alexa Fluor 488-conjugated donkey anti-rabbit IgG (Invitrogen, La Jolla, CA), for 2 hr at room temperature. Stained sections were mounted onto microscopy slides and visualized by confocal microscopy (Zeiss 510 META).

### Metabolic parameters

Body composition and locomotor activity of ten- to twelve-month old of AC3^−/−^ and AC3^+/+^ mice were measured as previously described [11]. Body composition was determined in conscious mice by quantitative magnetic resonance (QMR) (EchoMRI 3-in-machine Whole Body Composition Analyzer; Echo Medical Systems, Houston, TX). We measured locomotor activity in mice for 36 hrs. Locomotor activity was measured by the infrared beam break method using an Opto-Varimetrix-3 sensor system (Columbus Instruments). Locomotor activities of young animals were measured as previously described [12]. Mice were individually housed in rat cages for 3 days to allow them to adjust to the environment before recording voluntary activity. Movement episodes were recorded as infrared beam breaking. The recording was collected with the use of QA-4 input modules coupled with infrared motion detectors. Data was collected by VitalView Data Acquisition System (Mini Mitter, version 4.1) and transferred to the ActiView Biological Rhythm Analysis program (Mini Mitter, version 1.2) for actogram generation.

### Measurement of serum parameters

Blood was collected from mouse-tail veins and serum FFA levels were determined by a NEFA kit (Wako Diagnostics, Richmond, VA) according to the instructions of the manufacturer. We assayed serum triglyceride and glycerol by a serum triglyceride determination kit (Sigma, St. Louis, MO). Leptin and insulin levels were assayed with an ELISA kit (Crystal Chem Inc., Downers Grove, IL) according to the manufacturer's instructions. For *in vivo* study of β-AR agonists, CL316,243 (CL) (1 mg/kg), isoproterenol (0.1 mg/kg), or saline were injected intraperitoneally into AC3^+/+^ or AC3^−/−^ mice. Blood was collected 15 min later.

### Effects of leptin treatment on food intake and body weight

Mice were housed individually for at least one week to allow them to adjust to the new environment before starting the experiments. The food intake and body weight were measured daily for one week before vehicle injection. Vehicle (10 µl 0.9% NaCl/g body weight) was injected intraperitoneally twice (6:00 pm and 12:00 am) daily for two consecutive days. After one day of vehicle treatment, leptin (2 µg/g body weight) was injected intraperitoneally twice (6:00 pm and 12:00 am) daily for two consecutive days. Food intake and body weight were measured daily during the study and continued for one week thereafter. The average food intake and body weight for each animal before vehicle treatment taken over five consecutive days was taken as the basal value. We used the averages of food intake and body weight during vehicle treatment as the control for leptin effects.

### Statistical analysis

All data are expressed as means±SEM, unless otherwise indicated. We performed statistical analysis using the unpaired Student's t test. P<0.05 was considered to be statistically significant. For growth curves, the data were analyzed with two-way ANOVA (PRISM Version 4.0b).

## Results

### AC3^−/−^ mice are obese

**Figure 1:**
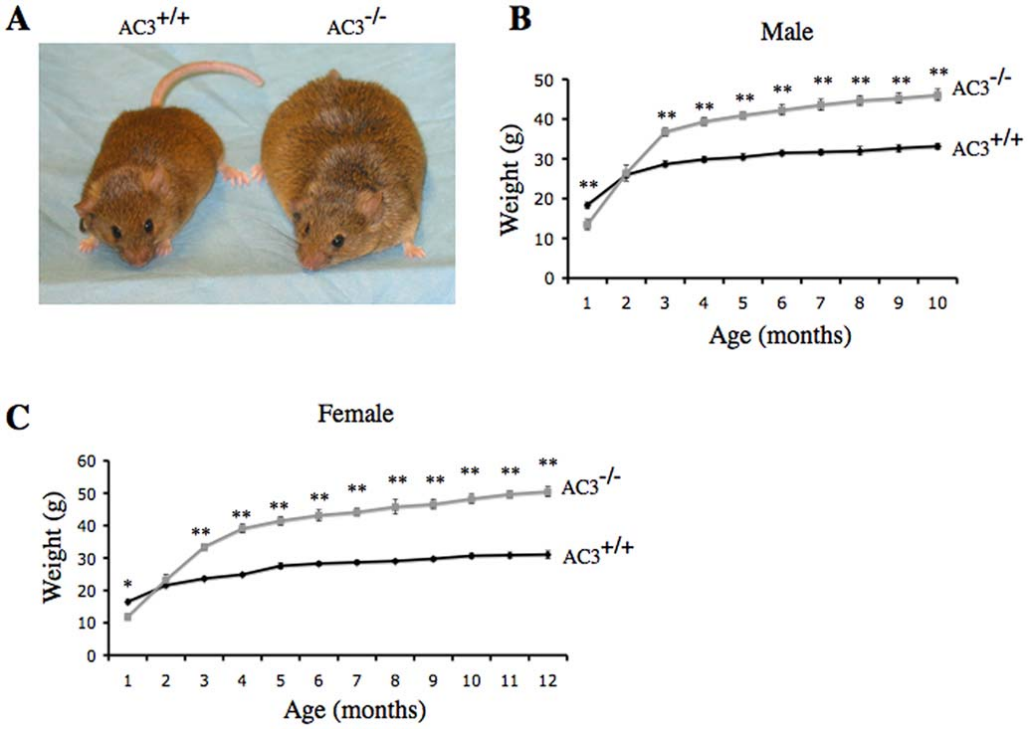
AC3^−/−^ mice exhibit adult onset obesity. (A) Representative eight-month-old AC3^+/+^ and AC3^−/−^ male mice. (B) The growth curve of AC3^−/−^ male mice compared to AC3^+/+^ male mice. (C) The growth curve of AC3^−/−^ female mice compared to AC3^+/+^ female mice. At each time point, the data are the average of 16 mice. Data are represented as means±SEM. *, p<0.05; **, p<0.01.

To investigate the importance of AC3 for regulation of body weight, we monitored the weight of AC3^−/−^ mice and wild type littermates over 12 months (Figure 1). During this period, mice were fed *ad libitum* on laboratory mouse chow. Although AC3^−/−^ mice are about half the size of their wild type littermates right after birth, they achieve normal size and weight after two months [9]. This smaller size during the first couple of months is attributable to their lack of olfaction and pheromone responses which impair suckling [9], [13]. As the mice age, however, they become obese and are significantly heavier than wild type littermates (Figure 1A). Adult male AC3^−/−^ mice are about 40% heavier than wild type male mice (Figure 1B) while female AC3^−/−^ are 70% heavier (Figure 1C).

**Figure 2:**
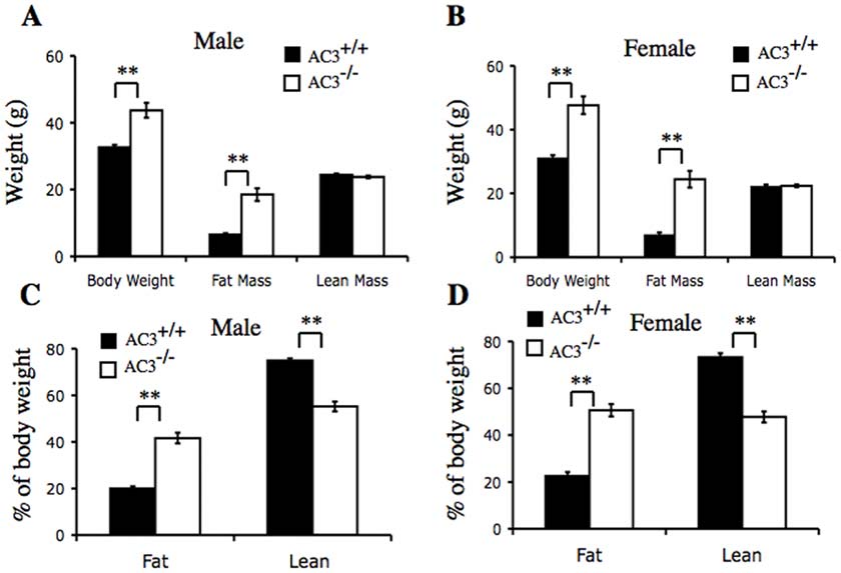
AC3^−/−^ mice weight gain is due to an increase in fat mass. The body compositions of AC3^+/+^ and AC3^−/−^ mice were determined in conscious mice by quantitative magnetic resonance (QMR). (A) Body composition of male AC3^+/+^ and AC3^−/−^ mice at 10 months. (B) Body composition of female AC3^+/+^ and AC3^−/−^ mice at 10 months. (C) Percentage of fat mass and lean mass of male AC3^+/+^ and AC3^−/−^ mice at 10 months. (D) Percentage of fat mass and lean mass of female AC3^+/+^ and AC3^−/−^ mice at 10 months. N = 8 mice for each group. Data are represented as means±SEM. **, p<0.01.

AC3^−/−^ mice were analyzed by quantitative magnetic resonance to determine body composition. A comparison of AC3^−/−^ male mice and wild type littermates indicates that the weight gain is exclusively in fat mass with no change in lean mass (Figure 2A). Fat mass comprised 41% of the weight of AC3^−/−^ male mice compared to 19% of the weight of AC3^+/+^ male mice (Figure 2C). Similarly, the weight difference between female AC3^−/−^ and AC3^+/+^ mice was attributable to a large increase in fat mass (Figure 2B). Fat accounted for 50% of the weight of female AC3^−/−^ mice and 23% for AC3^+/+^ females (Figure 2D).

**Figure 3:**
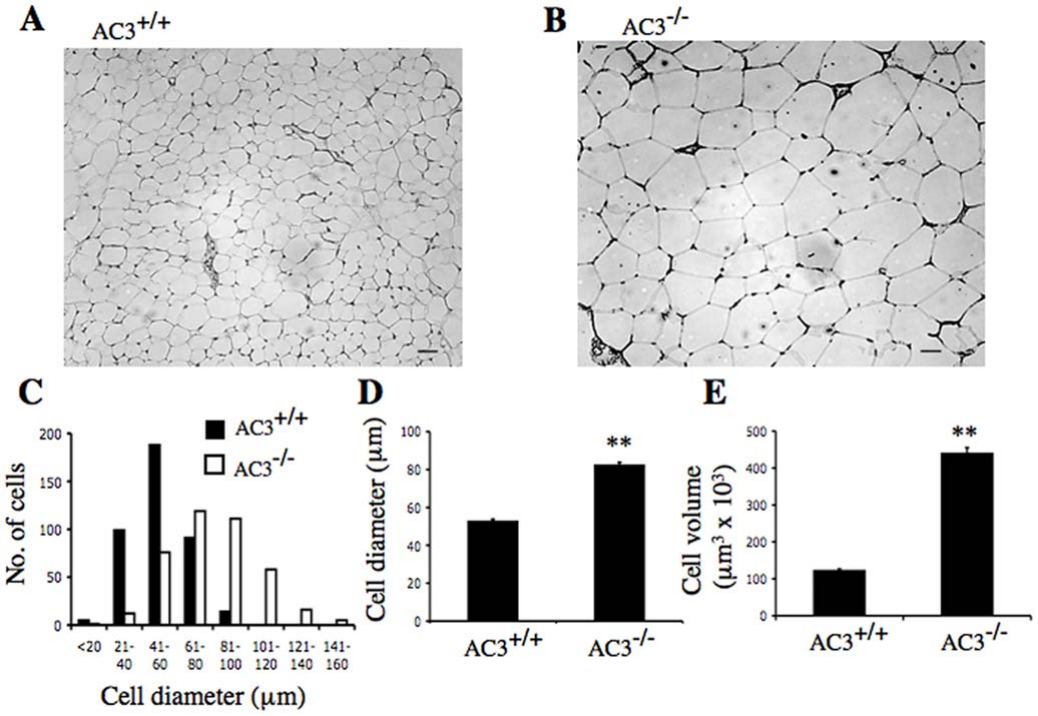
AC3^−/−^ mice have larger adipocytes. Representative hematoxylin sections of epididymal adipose tissue from (A) AC3^+/+^ and (B) AC3^−/−^ mice. Scale bar, 50 µm. (C) The distribution of adipocyte cell diameter of AC3^+/+^ and AC3^−/−^ mice. (D) The average adipocyte cell diameter of AC3^+/+^ and AC3^−/−^ mice. (E) The adipocyte cell volume of AC3^+/+^ and AC3^−/−^ mice. Data is the average from four AC3^−/−^ and four AC3^+/+^ mice at 8 months. A total of 400 cells from each genotype were counted. Data are represented as means±SEM. **, p<0.01.

Since obesity is often associated with adipocyte enlargement [14], [15], sections from epididymal adipose tissue of AC3^−/−^ and AC3^+/+^ mice were compared (Figure 3). The adipocytes from AC3^−/−^ mice were considerably larger than those from AC3^+/+^ mice (Figure 3A and 3B). This difference is reflected in the cell diameter (Figure 3C and 3D) as well as the cell volume which is approximately 3.5 times larger in AC3^−/−^ mice (Figure 3E). These data are consistent with the increased fat mass of AC3^−/−^ mice.

**Figure 4:**
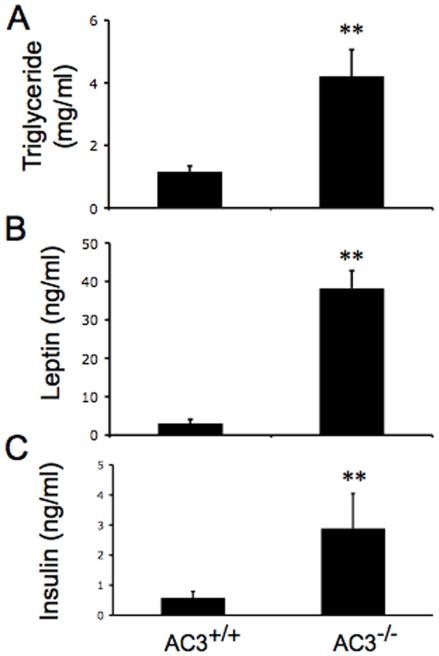
Serum leptin, insulin, and triglyceride are increased in AC3^−/−^ mice. (A) Serum triglyceride was measured in AC3^+/+^ and AC3^−/−^ mice at age of 8 months. (B) Serum leptin was measured in AC3^+/+^ and AC3^−/−^ mice at an age of 8 months. (C) Serum insulin was measured in AC3^+/+^ and AC3^−/−^ at 8 months. There were significant differences in triglyceride, leptin, and insulin levels between AC3^+/+^ and AC3^−/−^ mice. Each genotype of group mice consisted of 8 animals. Data are represented as means±SEM. **, p<0.01.

A key feature of obesity is increased production of triglycerides. Therefore, we monitored triacylglycerol levels to determine if AC3^−/−^ mice synthesize more triglyceride than wild type mice. Serum triglyceride levels in AC3^−/−^ mice and AC3^+/+^ mice were 4.2**±**0.1 mg/ml and 1.1**±**0.06 mg/ml, respectively (Figure 4A).

Leptin is an adipokine synthesized in adipose that is thought to be a hormonal indicator of fat accumulation. Since the expression and release of leptin depends on adipocyte size [16] and are often higher in obese patients [17], we compared serum leptin levels between AC3^−/−^ and AC3^+/+^ mice. Serum leptin is approximately 10 fold higher in AC3^−/−^ mice compared to AC3^+/+^ mice (Figure 4B). The difference in serum leptin between AC3^−/−^ and AC3^+/+^ was even greater when mice were treated with CL, a ß-3 adrenergic agonist or in fasting mice (data not shown**)**. Leptin increases in adult AC3^−/−^ mice may reflect increased adipocyte size and obesity.

Insulin is another adiposity signal that is secreted in proportion to adipocyte size and positively correlates with body weight [18], [19]. Consequently, we also measured serum insulin in adult AC3^−/−^ mice and their wild type littermates. Serum insulin levels in AC3^−/−^ mice were about 4 fold higher than wild type mice (Figure 4C). Insulin levels were also greater in AC3^−/−^ mice compared to AC3^+/+^ mice when the mice were treated with CL, or in fasting mice (data not shown).

### AC3^−/−^ mice show normal ß-adrenergic stimulated lipolysis

In white adipose, lipolysis is stimulated through ß-adrenergic receptors that are coupled to activation of adenylyl cyclase. Increases in cAMP stimulate cAMP-dependent protein kinase (PKA) which activates lipase, the enzyme that catalyzes the breakdown of triacylglycerol into glycerol and free fatty acids (FFA) [20]. Furthermore, adipose tissue has been reported to express mRNA for AC3 [21], [22]. Accordingly, we suspected that the obesity of AC3^−/−^ mice might be due to decreased lipolysis. To address this issue, lipolysis was measured by monitoring the increase in serum glycerol and FFA in AC3^+/+^ and AC3^−/−^ mice after i.p. administration of isoproterenol, a ß-adrenergic agonist. Surprisingly, there were no significant differences in isoproterenol-stimulated lipolysis between AC3^−/−^ and AC3^+/+^ mice (Figure S1). There was also no difference in isoproterenol-stimulated lipolysis when isoproterenol was varied over a broad range of concentrations up to 100 µg/kg, when CL was administered i.p., or with fasting animals (Figure S2). In addition, Western analysis indicated that AC3 protein is not expressed in white adipose (data not shown). However, low levels of AC3 protein were detected in brown adipose (Figure S3A), consistent with reports for the presence of AC3 mRNA in brown adipose [22]. Nevertheless, adenylyl cyclase activity in brown adipose from AC3^−/−^ and AC3^+/+^ mice was comparable (Figure S3B). Collectively, these data indicate the obesity of AC3^−/−^ mice is not due to a deficiency in ß-adrenergic stimulation of lipolysis.

### AC3^−/−^ mice consume more food and are less active than AC3^+/+^ mice

The obesity of AC3^−/−^ mice suggests that they either eat more food or are less active. Since AC3 is expressed in the hypothalamus [23] and food intake may be regulated by cAMP in the hypothalamus [24], we monitored the food consumption of AC3^−/−^ mice and their wild type littermates at an age of two months. At two months, AC3^−/−^ and AC3^+/+^ mice are of comparable weight.

**Figure 5:**
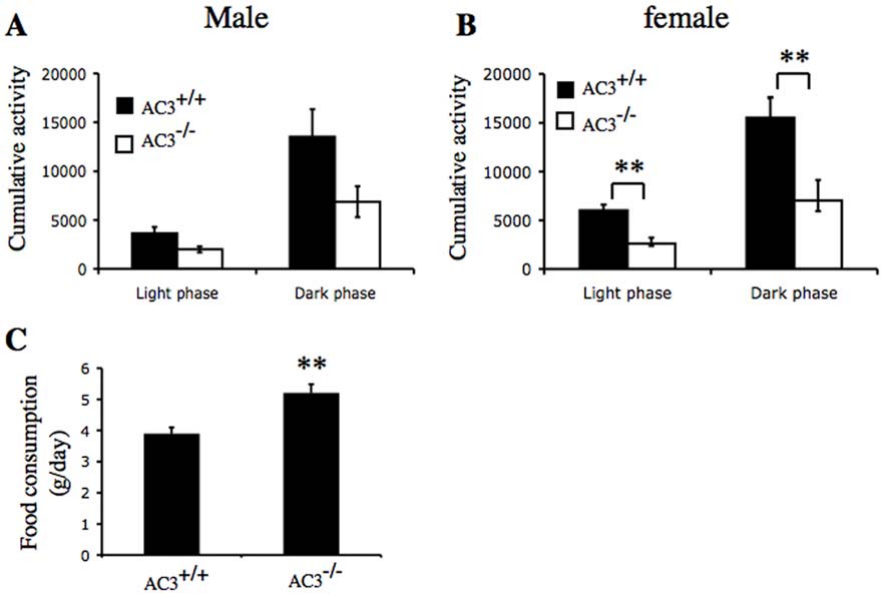
Adult AC3^−/−^ mice exhibit decreased locomotor activity. (A) Locomotor activity of male AC3^+/+^ and AC3^−/−^ mice. (B) Locomotor activity of female AC3^+/+^ and AC3^−/−^ mice. N = 8 animals for each group at an age of 10 months. (C) Food consumption of male AC3^+/+^ and AC3^−/−^ mice. N = 5 animals for each group at an age of 2 months. Data are represented as means±SEM. **, p<0.01.

**Figure 6:**
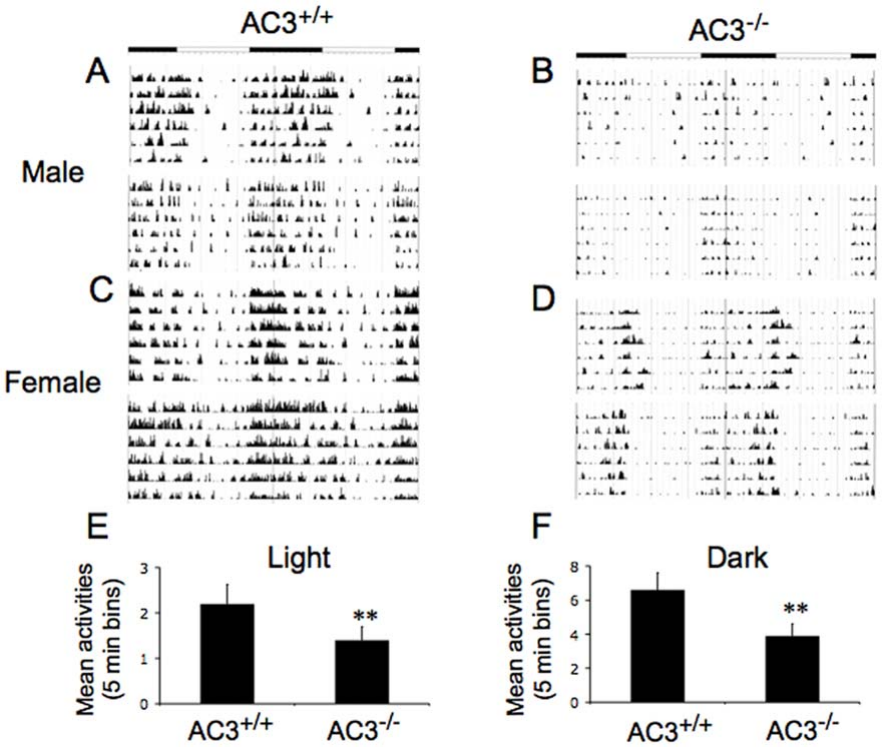
Young AC3^−/−^ mice demonstrate decreased locomotor activity. (A,B,C,D) Representative locomotor activity of two mice for 6 days: AC3^+/+^ male mice (A), AC3^−/−^ male mice (B), AC3^+/+^ female mice (C), and AC3^−/−^ female mice (D). (E) Average activity of AC3^+/+^ and AC3^−/−^ mice in the light phase. (F) Average activities of AC3^+/+^ and AC3^−/−^ mice in the dark phase. For (E,F), N = 12 mice at an age of 2 months. There is a significant difference in locomotor activity between AC3^+/+^ and AC3^−/−^ mice in the light and dark phases. **, p<0.01.

The daily food intake of AC3^−/−^ mice was 30% greater than AC3^+/+^ mice (Figure 5C) suggesting that increased food consumption may contribute to the obesity of AC3^−/−^ mice. Not surprisingly, AC3^−/−^ mice at an age of 10 months were less active than AC3^+/+^ mice (Figure 5A and 5B). Although lower physical activity is strongly associated with weight gain, it can be argued that low physical activity at 10 months is a consequence rather than a contributor to obesity [25]. Therefore, we also examined the activity of AC3^−/−^ mice before they exhibited obesity, at an age of two months (Figure 6). Cumulative activity of young adult AC3^−/−^ males and females was significantly less than AC3^+/+^ mice during the dark and light phase of the circadian cycle. These data indicate that AC3^−/−^ mice are obese because they eat more and are less active.

### Young AC3^−/−^ mice have elevated serum leptin levels

**Figure 7:**
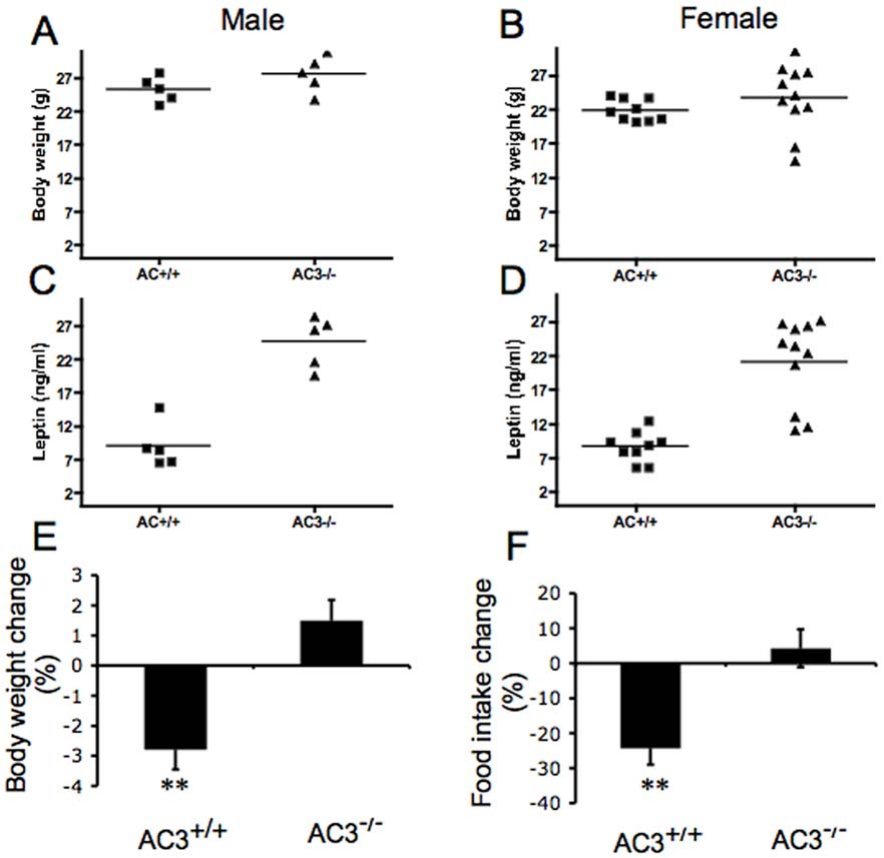
Young AC3^−/−^ mice have elevated serum leptin levels and low leptin sensitivity. (A) The body weight of young AC3^+/+^ and AC3^−/−^ male mice. N = 5 for each group of animals. There is no significant difference between AC3^+/+^ and AC3^−/−^ mice. (B) The body weight of young AC3^+/+^ and AC3^−/−^ female mice. For AC3^+/+^ female mice, N = 9; for AC3^−/−^ female mice, N = 11. There is no significant difference between AC3^+/+^ and AC3^−/−^ mice. (C) The serum leptin levels of young AC3^+/+^ and AC3^−/−^ male mice. N = 5 for each group. There is significant difference between AC3^+/+^ and AC3^−/−^ mice (p<0.01). (D) The serum leptin levels of young AC3^+/+^ and AC3^−/−^ female mice. For AC^+/+^ female mice, N = 9; for AC3^−/−^ female mice, n = 11. There is a significant difference between AC3^+/+^ and AC3^−/−^ mice (p<0.01). (E) The leptin effects of body weight in AC^+/+^ and AC3^−/−^ mice. N = 11 for each group of animals. There is a significant difference between AC3^+/+^ and AC3^−/−^ mice (p<0.01). (F) The leptin effects of food intake in AC^+/+^ and AC3^−/−^ mice. Each genotype of mice consists of 11 animals. There is a significant difference between AC3^+/+^ and AC3^−/−^ mice. **, p<0.01.

Although adult AC3^−/−^ mice exhibited elevated leptin levels (Figure 4B), it is generally thought that leptin resistance is a consequence of obesity, rather than a cause. However, it has been recently proposed that elevated leptin levels may cause obesity [26], [27], [28]. Accordingly, we measured serum leptin levels in AC3^−/−^ mice and their wild type littermates at two months. At this age, there were no significant differences in body weight between AC3^−/−^ mice and their wild type littermates (male AC3^+/+^ mice and AC3^−/−^ mice: 25.3±0.9 g and 27.6±1.2 g, respectively; female AC3^+/+^ mice Vs AC3^−/−^ mice: 21.9±0.5 g and 23.8±1.5 g, respectively, Figure 7A and 7B). However, the serum leptin levels in AC3^−/−^ mice were significantly higher than AC3^+/+^ mice (male AC3^+/+^ mice and AC3^−/−^ mice: 9±1.5 ng/ml and 24.6±1.7 ng/ml, respectively; female AC3^+/+^ mice and AC3^−/−^ mice: 8.7±0.7 ng/ml and 21.1±1.9 ng/ml, respectively, Figure 7C and 7D).

In normal mice, leptin signals the central nervous system to reduce food intake [29], and administration of leptin causes a weight loss [30], [31], [32]. Leptin-resistant obese animals and patients are weakly responsive or unresponsive to exogenously administrated leptin. To determine whether AC3^−/−^ mice are leptin resistant, leptin was administered to male AC3^−/−^ and wild type mice twice daily (i.p., 2 µg/g body weight). As expected, the body weight and food intake of AC3^+/+^ mice decreased after two days of leptin treatment (Figure 7E and 7F). In contrast, food intake of AC3^−/−^ mice was unaffected by administration of leptin and these mice continued to gain weight (Figure 7E and 7F). These data suggest that the increased food intake and obesity of AC3^−/−^ mice may be due to leptin resistance.

### Adenylyl cyclase activity in the hypothalamus is reduced in AC3^−/−^ mice

**Figure 8:**
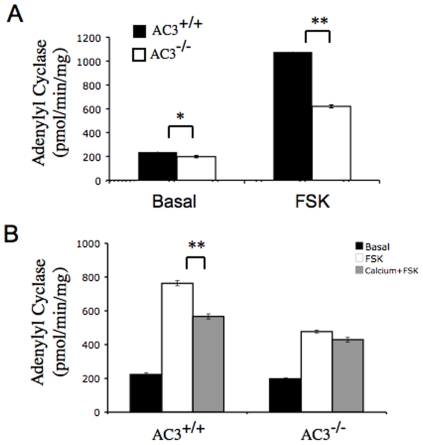
The adenylyl cyclase activity of the hypothalamus is reduced in AC3^−/−^ mice. (A) Forskolin stimulated adenylyl cyclase activity in the hypothalamus is absent in AC3^−/−^ mice. (B) Calcium inhibition of forskolin-stimulated adenylyl cyclase in the hypothalamus is impaired in AC3^−/−^ mice. The adenylyl cyclase activity was assayed from homogenized hypothalamus. The forskolin concentration was 50 µM. The free calcium concentration was 19.9 µM. Data are represented as means±SEM. *, p<0.05; **, p<0.01. N = 3 for each genotype.

We measured adenylyl cyclase activity in the hypothalamus of AC3^−/−^ mice because it plays a major role in appetite and weight control. Most mammalian cells contain a mixture of adenylyl cyclases. The most reliable measure of total adenylyl cyclase activity, forskolin-stimulated activity, was reduced 40% in the hypothalamus of AC3^−/−^ mice compared to wild-type littermates (Figure 8A). Since AC3 is a Ca^2+^-inhibted adenylyl cyclase [33], adenylyl cyclase activity was also assayed in the presence of Ca^2+^ to confirm the presence of AC3 in the hypothalmus. Forskolin-stimulated adenylyl cyclase activity in the hypothalamus of wild type was significantly inhibited by Ca^2+^. However, Ca^2+^ did not inhibit adenylyl cyclase activity in the hypothalmus of AC3^−/−^ mice (Figure 8B). Collectively, these data indicate that AC3 is expressed in the hypothalmus and that it accounts for all of the Ca^2+^-inhibted adenylyl cyclase activity in the hypothalamus.

### AC3 is expressed in primary cilia of hypothalamic neurons

**Figure 9:**
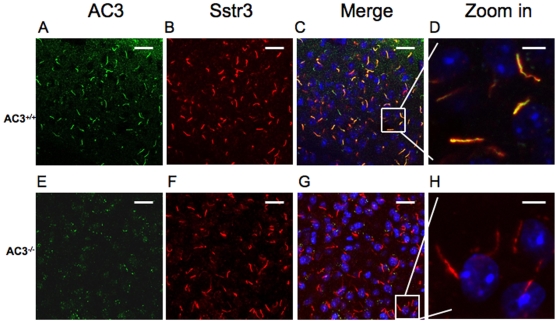
AC3 is expressed in the cilia of VMH neurons. (A,B,C,D) Representative images of AC3 immunoreactive cilia in the VMH regions of the hypothalamus in AC3^+/+^ mice labeled with antibodies AC3 (green) (A), Sstr3 (red) (B) and colocalization of AC3 and Sstr3 (merge). Scale bar, 20 µm. (E,F,G,H) Representative images of immunoreactive cilia in the VMH regions of the hypothalamus in AC3^−/−^ mice labeled with antibodies AC3 (green) (E), Sstr3 (red) (F) and colocalization of AC3 and Sstr3 (merge). Scale bar, 20 µm. (D,H) Enlarged images of selected areas in of AC3^+/+^ (D) and AC3^−/−^ (H). Scale bar, 5 µm. Nuclei are stained with Hoestch (blue). N = 3 for each genotype.

Primary cilia, the non-motile cilia, are microtubule-based organelles that project from the basal bodies of many cells, including neurons in the hypothalamus. There is a strong correlation between defects in proteins found in primary cilia of hypothalamic neurons and obesity [34], [35], [36]. Therefore, we stained hypothalamic sections with an AC3 antibody to determine if the enzyme is expressed in primary cilia of the hypothalamus. The primary cilia in the hypothalamus stained positive for AC3, and AC3 was especially abundant in the ventral medial hypothalamus (VMH) region (Figure 9A). However, AC3^−/−^ mice showed no AC3-positive cilia (Figure 8E). Sections from the hypothalamus were also labeled with antibodies against Sstr3, another primary cilia marker [37], [38]. Costaining with AC3 and Sstr3 antibodies in wild type mice showed colocalization of AC3 and Sstr3, confirming the presence of AC3 in primary cilia. Although sections from AC3^−/−^ mice were negative for AC3, there were no obvious differences in the appearance of primary cilia, suggesting that AC3 may not be required for the structural integrity of primary cilia (Figure 8B and 8F). The presence of AC3 in primary cilia of the hypothalamus suggests the interesting possibility that the leptin insensitivity of AC3^−/−^ mice may be due to defects in signaling events in primary cilia that depend on cAMP signals generated by AC3.

## Discussion

AC3 gene polymorphisms are associated with obesity in humans suggesting that AC3 may play a role in weight regulation [8], a hypothesis that is strengthened by the presence of AC3 in the hypothalamus. The objective of this study was to determine if disruption of the gene for AC3 in mice causes obesity. We have discovered that AC3^−/−^ mice exhibit pronounced obesity which is due primarily to higher fat mass compared to their wild type littermates. They also exhibit higher levels of leptin as well as leptin resistance, both of which are commonly associated with obesity. Furthermore, AC3^−/−^ mice are less active throughout their circadian cycle, and they consume more food. The decreased locomotor activity, greater food consumption, and leptin resistance exhibited by AC3^−/−^ mice are not caused by obesity since these changes are seen in young, non-obese AC3^−/−^ mice.

Since AC3 is expressed in adipose tissue, an obvious explanation for the obesity of AC3^−/−^ mice would be a deficiency in lipolysis. However, AC3^−/−^ mice exhibit normal ß-adrenergic stimulation of lipolysis. Most importantly, we were unable to detect AC3 protein in white adipose by Western analysis, although it was present in brown adipose. Consequently, the obesity of AC3^−/−^ mice is not due to a loss of AC3 from white adipose and a decrease in lipolysis; it is most likely due to defects in cAMP signaling within the hypothalamus.

One of the physiological roles of leptin is to signal the nutritional status of the animal to the brain [39]. When there is an excess of triacylglycerol stored in adipose, leptin is secreted to reduce appetite and promote energy expenditure. Obesity is often associated with increased leptin production and leptin insensitivity [40]. It has been generally thought that leptin resistance is a consequence of obesity. However, new evidence shows that elevated leptin levels in the circulatory system contribute to obesity [27]. For example, transgenic mice overexpressing leptin exhibit adult-onset obesity [41], and they are more susceptible to diet-induced obesity because of increased leptin secretion [42]. Furthermore, chronic leptin treatment in diet-induced obese rats accelerates dietary obesity [43]. Moreover, wheel running synergizes with leptin treatment markedly to reduce body weight in obese animals induced by a high-fat diet, even though neither reduce body weight alone [43]. Therefore, we believe that the obesity of AC3^−/−^ mice is caused by a combination of lower physical activity, hyperphagia, and decreased leptin sensitivity.

AC3 protein is widely distributed throughout the hypothalamus with especially high levels in the VMH. The VMH is the “satiety center” of the hypothalamus [44], and lesions of the VMH cause obesity because of increased food consumption and low physical activity [44], [45]. Furthermore, transgenic mice lacking specific proteins expressed in the VMH including leptin receptor, steroidogenic factor 1 (SF-1), melanocortin-4 receptor (MC4R), and brain-derived neurotrophic factor (BDNF), exhibits obesity, hyperphagia, and low locomotor activity similar to AC3^−/−^ mice [46], [47], [48], [49], [50], [51]. Although feeding behavior and leptin signaling may be regulated by cAMP levels in the hypothalamus [52], [53], the mechanism by which cAMP signals in the hypothalamus regulates feeding behavior or leptin actions is unknown. Nevertheless, the cyclic AMP (cAMP) responsive element-binding protein-1 (CREB1) is expressed in the VMH [54], and CREB1^−/−^ mice exhibit adult onset obesity associated with hyperphagia, elevated insulin and leptin levels, as well as leptin resistance [54]. Furthermore, there is a negative correlation between cAMP synthesis and food intake [55]. Food deprivation decreases cAMP levels in the VMH and dorsomedial hypothalamus (DMH), whereas refeeding normalizes it, indicating that the cAMP-induced anorexia is due to cAMP mediated signaling in the VMH and DMH [55]. Our data suggests that AC3 in the hypothalamus may generate cAMP which limits food consumption and regulates leptin signaling.

During evolution, multiple mechanisms have been conserved to control body weight in animals. There is considerable evidence that cAMP plays an important role in regulation of energy balance in mammals [52] and there are a large number of targets downstream of cAMP that have the potential to play an important role in regulation of body weight [53]. Although the mechanisms by which hypothalamic cAMP regulates food intake, physical activity and leptin sensitivity are not know, our study identifies AC3 in the primary cilia of the hypothalamus as a critical source of cAMP important for regulation of these processes.

One of the most striking things about the distribution of AC3 in neurons of the VMH is the fact that it is localized almost exclusively to primary cilia. Although the function of these structures in hypothalamic neurons is not known, it is interesting that there are a number of human diseases and transgenic mice in which alterations in components of the primary cilia are associated with obesity. For example, Bardel-Biedl syndrome (BBS) is a pleiotropic disorder characterized by a multitude of symptoms, including obesity [56]. BBS is due to disruption of the cilia/basal body [56], [57]. Alstrom syndrome is a rare autosomal recessive disorder characterized by obesity, insulin resistance, and type 2 diabetes [58]. ALSM1 is also thought to be due to a defect in primary cilia [58]. The similarity between AC3^−/−^ and BBS^−/−^ mice is remarkable. Both animals exhibit obesity, hyperphagia, lower locomotor activity, and leptin resistance [28], [59]. AC3^−/−^ and BBS^−/−^ mice are also anosmic, and olfactory neurons in the main olfactory epithelium show negative electro-olfactogram responses [9], [13], [60]. Since AC3 and BBS proteins are localized to primary cilia, we speculate that the obesity phenotype exhibited by AC3^−/−^ mice may be due to disruption of cAMP signaling in primary cilia.

Single nucleotide polymorphisms (SNPs) in the intron1 and intron2 regions of AC3 gene are associated with obesity in Swedish man [8], a finding supported by our discovery that disruption of the AC3 gene in mice causes obesity. In addition, our preliminary data indicate that AC3^+/−^ mice may also become obese as they age, although their phenotype is weaker than AC3^−/−^ mice (data not shown). The discovery that AC3^−/−^ mice are obese emphasizes the importance of studying polymorphisms associated with human diseases and suggests that it may be interesting to explore the relationship between AC3 gene biomarkers and human obesity more fully.

In summary, the phenotype exhibited by AC3^−/−^ mice are consistent with the hypothesis that AC3 generates a cAMP signal in the primary cilia of the hypothalamus that is important for regulation of weight and leptin sensitivity. The phenotype of the AC3^−/−^ mouse is consistent with recent data implicating AC3 polymorphisms in obesity of humans.

## Supporting Information

Figure S1 Lipolysis is normal in AC3^−/−^ mice. (A) Serum glycerol and (B) Serum FFA levels in AC3^+/+^ and AC3^−/−^ mice were monitored 15 minutes after I.P. administration of isoproterenol (0.1 mg/kg body weight) or vehicle (0.9% sodium chloride). N = 6 mice per group.
(0.09 MB PPT)

Figure S2 AC3^−/−^ mice exhibit normal lipolysis. (A) Serum FFA levels for AC3^+/+^ and AC3^−/−^ mice during the fed state. (B) Serum FFA levels for the AC3^+/+^ and AC3^−/−^ mice during the fasted state. (C) Serum FFA levels for the AC3^+/+^ and AC3^−/−^ mice with injection of CL (0.1 mg/kg body weight). (D) Serum FFA levels for the AC3^+/+^ and AC3^−/−^ mice with injection of isoproterenol (ISO, 5 ng/kg body weight). N = 6 for each genotype. There is no significant difference in the serum FFA levels of AC3^+/+^ and AC3^−/−^mice treated with vehicle AC3^+/+^ mice: 0.39±0.1 mMol/L; AC3^−/−^ mice: 0.42±0.2 mMol/L; p>0.5). Data are represented as means±SEM. There is no significant difference in lipolysis between AC3^+/+^ and AC3^−/−^ mice.
(0.12 MB PPT)

Figure S3 The adenylyl cyclase activity of brown adipose tissue (BAT) is normal in AC3^−/−^ mice. (A) Representative western blots of BAT extracts from AC3^+/+^ and AC3^−/−^ mice. (B) The adenylyl cyclase activity of BAT stimulated by CL and norepinephrine (NE) in AC3^+/+^ and AC3^−/−^ mice. There is no significant difference in Cl or NE-stimulated adipose adenylyl cyclase activity from AC3^+/+^ and AC3^−/−^ mice. Each genotype of mice consists of 4 animals. The results are averaged of three experiments. Data are means±SEM.
(0.35 MB PPT)
