# Supplementary figures and images for: Adult Type 3 Adenylyl Cyclase–Deficient Mice Are Obese
Source: PLoS One. 2009 Sep 11;4(9):e6979. doi: 10.1371/journal.pone.0006979 (PMC2735775; doi:10.1371/journal.pone.0006979)

## Slide 1
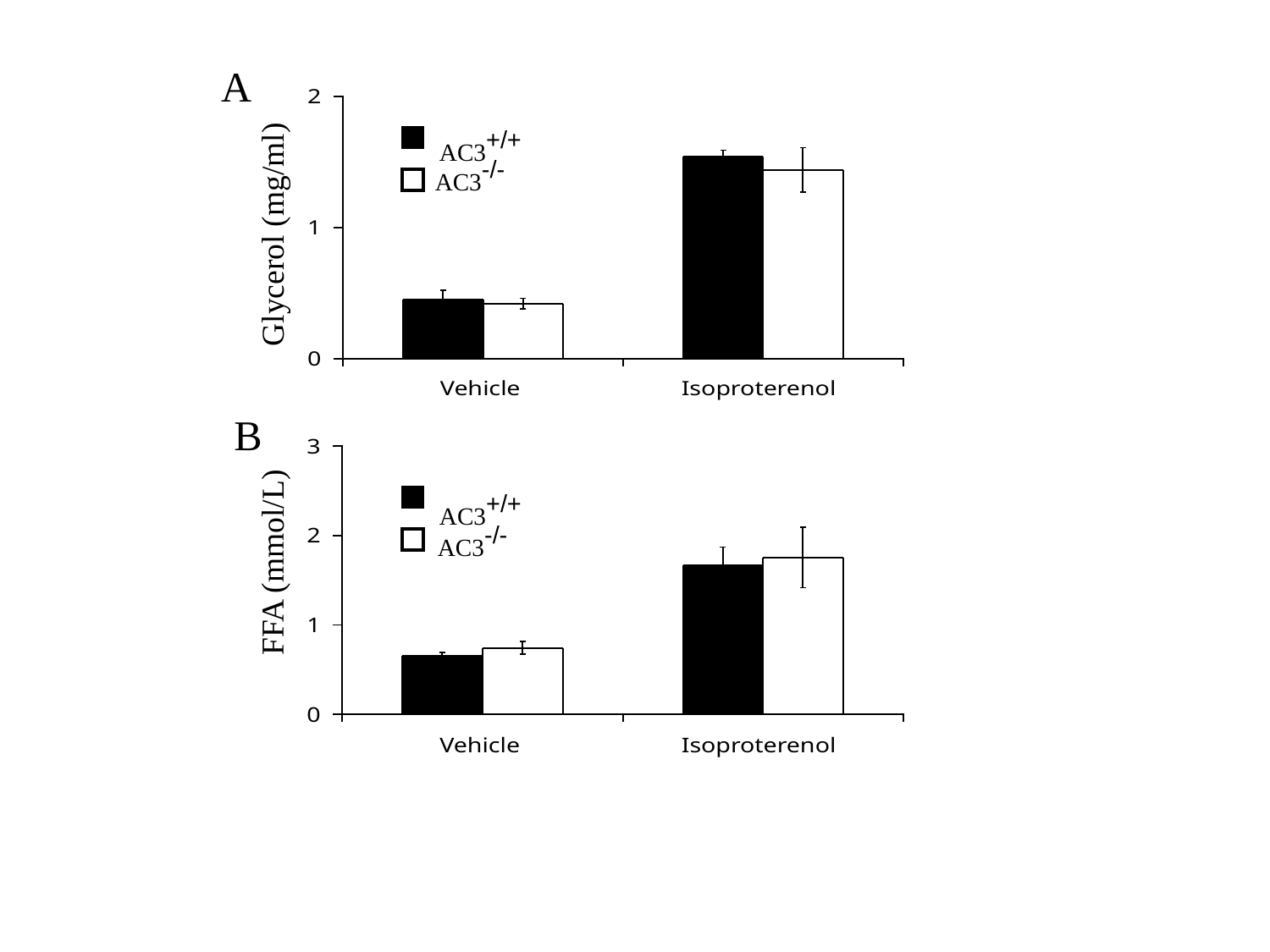

A
AC3+/+
AC3-/-
Glycerol (mg/ml)
B
AC3+/+
AC3-/-
FFA (mmol/L)

Supplement: Figure S1 — Lipolysis is normal in AC3−/− mice. (A) Serum glycerol and (B) Serum FFA levels in AC3+/+ and AC3−/− mice were monitored 15 minutes after I.P. administration of isoproterenol (0.1 mg/kg body weight) or vehicle (0.9% sodium chloride). N = 6 mice per group. (0.09 MB PPT) [file pone.0006979.s001.ppt]

## Slide 1
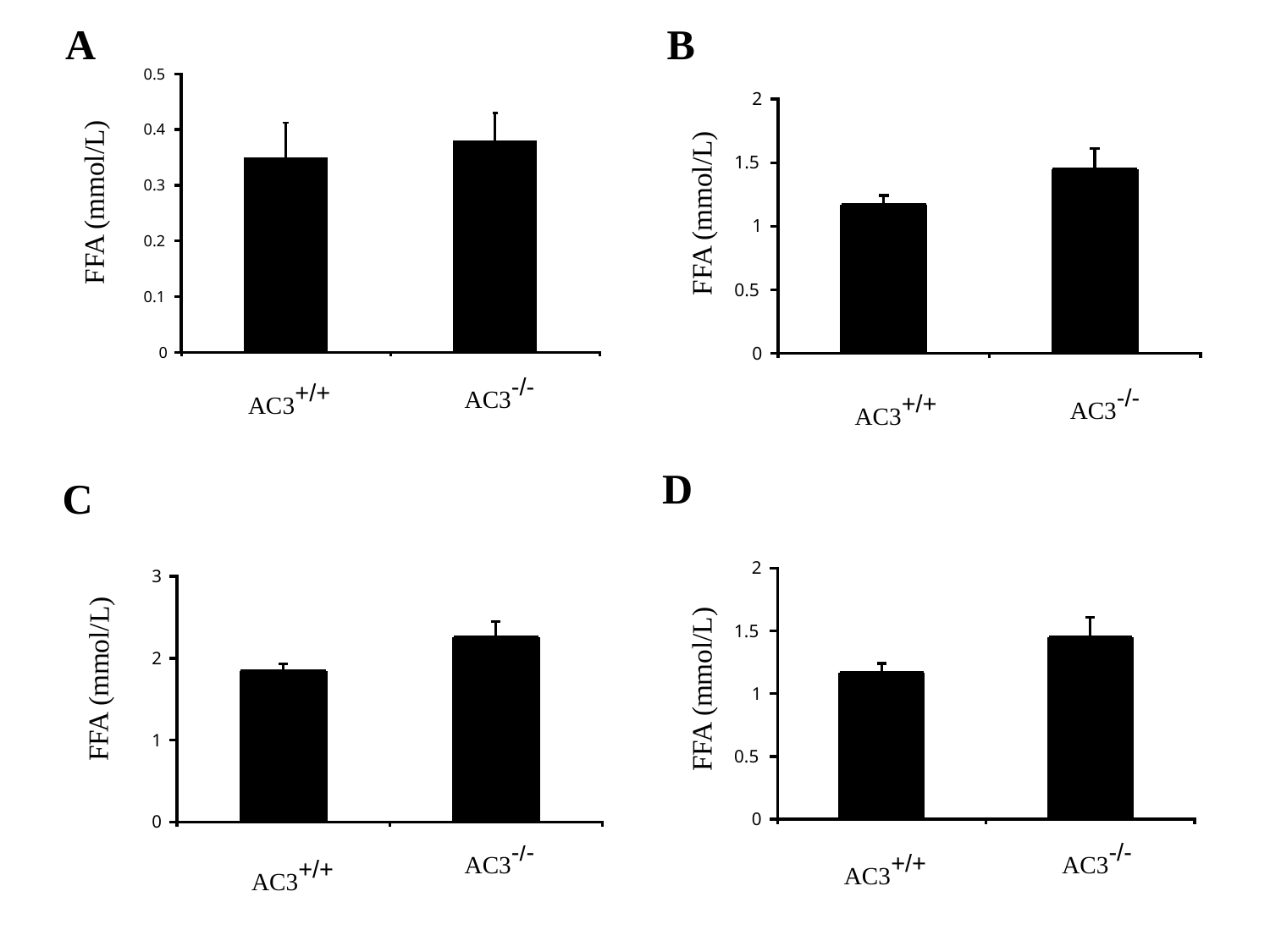

A
B
FFA (mmol/L)
FFA (mmol/L)
AC3-/-
AC3+/+
AC3-/-
AC3+/+
D
C
FFA (mmol/L)
FFA (mmol/L)
AC3-/-
AC3-/-
AC3+/+
AC3+/+

Supplement: Figure S2 — AC3−/− mice exhibit normal lipolysis. (A) Serum FFA levels for AC3+/+ and AC3−/− mice during the fed state. (B) Serum FFA levels for the AC3+/+ and AC3−/− mice during the fasted state. (C) Serum FFA levels for the AC3+/+ and AC3−/− mice with injection of CL (0.1 mg/kg body weight). (D) Serum FFA levels for the AC3+/+ and AC3−/− mice with injection of isoproterenol (ISO, 5 ng/kg body weight). N = 6 for each genotype. There is no significant difference in the serum FFA levels of AC3+/+ and AC3−/−mice treated with vehicle AC3+/+ mice: 0.39±0.1 mMol/L; AC3−/− mice: 0.42±0.2 mMol/L; p>0.5). Data are represented as means±SEM. There is no significant difference in lipolysis between AC3+/+ and AC3−/− mice. (0.12 MB PPT) [file pone.0006979.s002.ppt]

## Slide 1
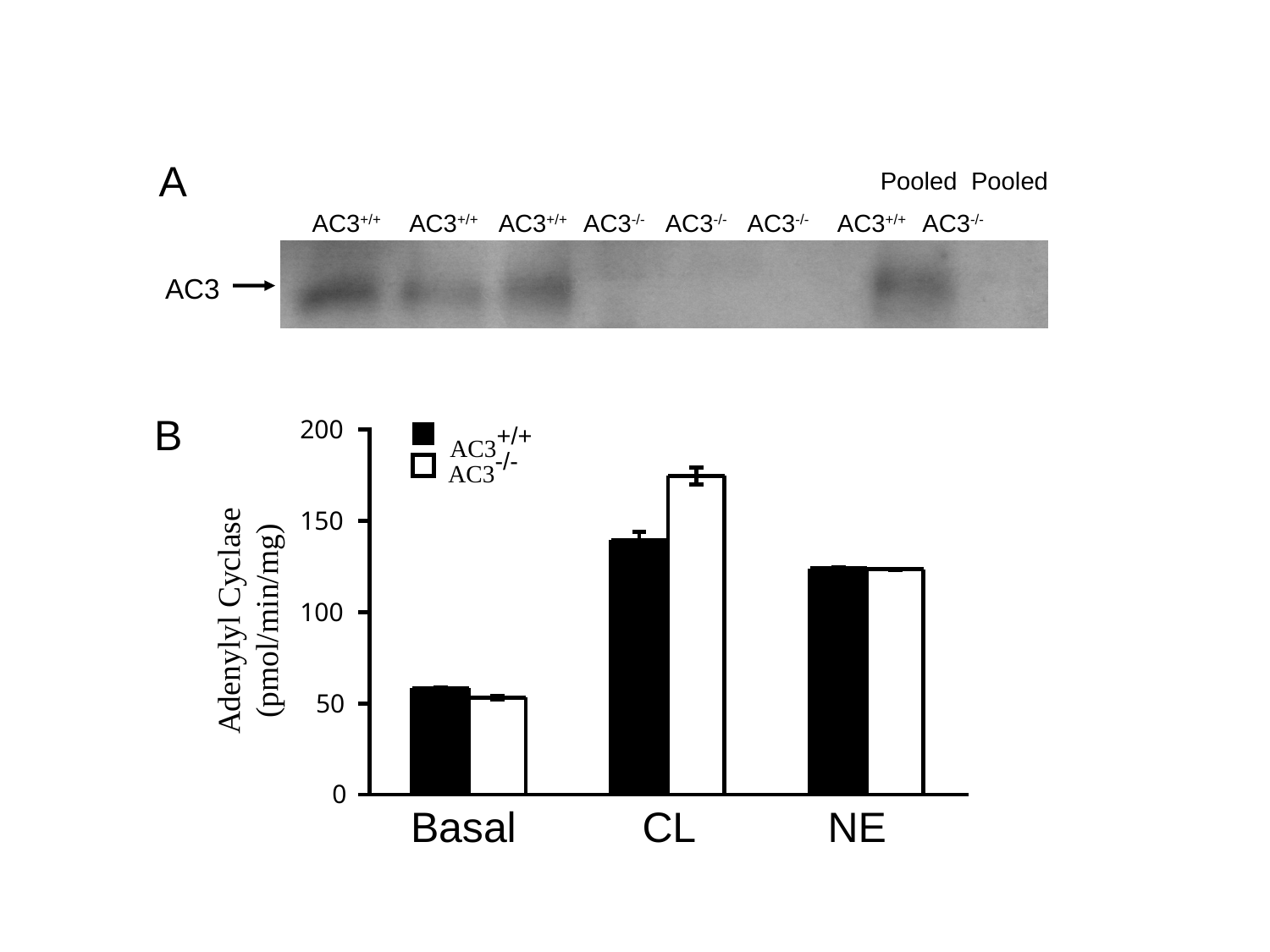

A
Pooled Pooled
 AC3+/+ AC3+/+ AC3+/+ AC3-/- AC3-/- AC3-/- AC3+/+ AC3-/-
AC3
B
AC3+/+
AC3-/-
Adenylyl Cyclase
(pmol/min/mg)
Basal
CL
NE

Supplement: Figure S3 — The adenylyl cyclase activity of brown adipose tissue (BAT) is normal in AC3−/− mice. (A) Representative western blots of BAT extracts from AC3+/+ and AC3−/− mice. (B) The adenylyl cyclase activity of BAT stimulated by CL and norepinephrine (NE) in AC3+/+ and AC3−/− mice. There is no significant difference in Cl or NE-stimulated adipose adenylyl cyclase activity from AC3+/+ and AC3−/− mice. Each genotype of mice consists of 4 animals. The results are averaged of three experiments. Data are means±SEM. (0.35 MB PPT) [file pone.0006979.s003.ppt]
